# How inclusive were UK-based randomised controlled trials of COVID-19 vaccines? A systematic review investigating enrolment of Black adults and adult ethnic minorities

**DOI:** 10.1186/s13063-024-08054-4

**Published:** 2024-04-12

**Authors:** Hibba Herieka, Daphne Babalis, Evangelia Tzala, Shyam Budhathoki, Nicholas A. Johnson

**Affiliations:** 1 University of Leicester Medical School, University of Leicester, Leicester, UK; 2 Imperial Clinical Trials Unit, School of Public Health, Faculty of Medicine, Imperial College London, Stadium House, 68 Wood Lane, London, W12 7RH, London, UK; 3 School of Public Health, Faculty of Medicine, Imperial College London, London, UK

## Abstract

**Objectives:**

To establish if Black adults and adult ethnic minorities, defined as any group except White British, were represented in UK-based COVID-19 vaccination randomised controlled trials (RCTs) when compared to corresponding UK population proportions, based on 2011 census data.

**Design:**

Systematic review of COVID-19 Randomised Controlled Vaccine Trials

**Setting:**

United Kingdom

**Participants:**

Randomised Controlled Trials of COVID-19 vaccines conducted in the UK were systematically reviewed following PRISMA guidelines. MeSH terms included “Covid-19 vaccine”, “Ad26COVS1”, and “BNT162 Vaccine” with keywords such as [covishield OR coronavac OR Vaxzevria OR NVX-CoV2373] also used. Studies that provided (A) participant demographics and (B) full eligibility criteria were included. The following key data was extracted for analysis: number of participants analysed, number of Black adults and number of adult minority ethnicity participants.

**Primary and Secondary Outcome Measures:**

The primary outcome is the mean percentage of Black adults randomised to COVID-19 vaccine trials deemed eligible within this review. The secondary outcome is the mean percentage of adult ethnic minorities randomised.

**Results:**

The final review included 7 papers and a total of 87 sets of data collated from trial sites across the UK. The standard mean percentage of Black adults included in the trials (0.59%, 95% CI: 0.13% - 1.05%) was significantly lower compared to the recorded Black adult population (2.67%) indicating that they were under-served in UK based COVID-19 vaccine RCTs (p < 0.001). Adult ethnic minority presence (8.94%, 95% CI: 2.07% - 15.80%) was also lower than census data (16.30%), indicating they were also under-served (p = 0.039).

**Conclusion:**

The findings show that COVID-19 vaccine trials failed to adequately randomise proportionate numbers of Black adults and adult minority ethnicities. More inclusive practices must be developed and implemented in the recruitment of underserved groups to understand the true impact of COVID-19.

**Supplementary Information:**

The online version contains supplementary material available at 10.1186/s13063-024-08054-4.

## Strengths and Limitations of this study

• The seven trials identified from the available literature allowed for a large sample of ethnicity data to be extracted from multiple sites for this review (*n*=20,439), improving its generalisability against the overall population.

• Due to the nature of the pandemic, multiple high-level RCTs were undertaken over a very short period, allowing a ‘snapshot’ of how inclusive trials were across this period and to investigate how effectively NIHR-INCLUDE guidance had been implemented.

• One limitation is that not all studies included recruited from the entirety of the UK. Some were exclusively recruiting from England (*n*=4), England and Wales (*n*=1), and England, Wales and Scotland (*n*=1).

• The use of 2011 census data as a comparator to determine inclusivity is likely to be an underestimate. However, it is currently considered as a gold standard in terms of providing figures for the UK adult ethnic profile, as well as for regional ethnic profiles. Therefore, its use is considered justified within the methodology.

## Introduction

Since its emergence in December 2019, the SARS-CoV-2 COVID-19 pandemic has resulted in over 6 million confirmed deaths worldwide [1]. This public health crisis prompted a race to develop an all-important vaccination - vital to vulnerable groups bearing the brunt of the disease burden [2]. However, it also highlighted how clinical research is not representative of the population it aims to serve [3]. This encompasses ethnic minorities, defined as any ethnic group except White British [4]. For example, Black British adults have an increased risk of death of up to 50% in comparison to their White counterparts when infected with COVID-19 [2]. Despite this, in 2020 the National Institute for Health and Care Research (NIHR) established ethnic minorities constituted only 9.26% of participants in NIHR-supported, UK COVID-19 studies, despite making up (based on NIHR figures) 13.80% of the UK population [3].

The increased threat of COVID-19 towards ethnic minorities and its adverse outcomes arises from a multitude of reasons [5]. Deprivation can exacerbate the effects of COVID-19 [6]. This encompasses house overcrowding, which is eight times more prevalent in Black African households than in White British households [7]. Proximity to infected individuals facilitates the droplet spread of COVID-19 [8], and overcrowded housing provides the appropriate setting for this. This can also be applied when considering where most ethnic minorities are based. Across England and Wales, London is the most densely populated and ethnically diverse region, where 40.2% of residents identified as either Asian, Black, Mixed or Other [9]. Popular urban areas like this create a desirable environment for the virus to spread easily, adding to the risk within these groups.

The risk associated with COVID-19 for ethnic minorities is also associated with lower vaccine uptake rates in comparison to their White counterparts [10]. In the UK, those with ethnicity defined as Black or Black British have the highest rates of vaccine hesitancy, with reasons cited for this being a lack of trust in the vaccine and worries about unknown side effects [11]. This, in turn, creates a vicious cycle, where distrust between ethnic minorities and vaccine manufacturers leads to lower rates of presentation for clinical trials and consequently, low inclusion of these groups [12].

Consequently, as minority ethnicities face greater risk with COVID-19, and that vaccine uptake is generally lower, it is important to ensure that minority ethnicities are represented within COVID-19 vaccine trials. However, underrepresentation in clinical trials compared to population estimates is an issue that not only affects ethnic minorities. Identified under-served groups include, but are not limited to: children, older people and patients with multimorbidity [13]. To address this issue, the NIHR launched the “Innovations in Clinical Trial Design and Delivery for the Under-served” roadmap, also known as the NIHR–INCLUDE [14] (often simply referred to as INCLUDE). INCLUDE is a strategic level overview to provide guidance in order to remove the lack to systemic approach in place to address inequality in research with an overall aim of making research more representative.

Its establishment in 2017 dictates that COVID-19 vaccination trials should be reflective of these guidelines. However, the degree to which this has been implemented is uncertain: no published peer-reviewed literature could be found during the literature search that focussed on the level of Black adult representation, and existing data is reflective of both adult and child ethnic minority participation across observational. interventional and non-randomised studies, as well as randomised clinical trials (RCTs) [3]. It is key to establish whether COVID-19 trials were representative to provide an indication as to whether the provision of INCLUDE [14] guidance was adequate to ensure inclusivity.

Based on the above, alongside the lack of current research on under-served ethnic representation, this study was conducted to investigate whether Black adults and adult ethnic minorities were represented in COVID-19 vaccine trials when comparing to the proportion they make up within the UK population.

## Method

### Study design

This is a systematic review with a meta-analysis that included published literature on UK-based COVID-19 vaccine RCTs.

### Outcome measures

We selected two outcome measures that helped us answer the question “How inclusive were UK-based randomised controlled trials of the COVID-19 vaccine?”:

- *Primary outcome measure:* Proportion of Black adults – defined as those identifying as Black, Black British, Caribbean or African - involved in COVID-19 vaccine RCTs. This is representative of the UK Black adult population
- *Secondary outcome measure:* Proportion of adult ethnic minorities – defined as any ethnic group except White British - involved in COVID-19 vaccine RCTs. This is representative of the UK adult ethnic minority population

These outcomes were achieved by assessing the participant demographics and eligibility criteria of the extracted publications. By performing a meta-analysis, extracted data was then compared against 2011 UK census data [15–17] to determine if studies were inclusive of the selected under-served groups.

### Literature Search

To perform this systematic review; OVID Medline, OVID Embase and The Cochrane Central Register of Controlled Trials were screened as per PRISMA guidelines [18].

Inclusivity of under-served groups in COVID-19 clinical trials was the initial outline of this review. Using the aforementioned databases and register, a search of the existing literature was performed for publications categorised under the Medical subject headings (MeSH) terms “Covid-19 vaccine”, “Ad26COVS1”, “BNT162 Vaccine” and “2019 ncov vaccine mRNA 1273”. A keywords search was also performed for terms such as [covishield OR coronavac OR Vaxzevria OR NVX-CoV2373 OR elasomeran OR azd1222 OR comirnaty] included in the title or abstract of available studies. This search yielded a total of 46,082 results. Particular under-served groups identified were Black adults and adult ethnic minorities, leading to the focus of this report.

The type of clinical trial investigated in this systematic review is RCTs. This decision was supported by the clear and consistent structure of both the methodology and results displayed within RCTs, meaning that there will be homogeneity in the data retrieved for meta-analysis. RCTs are also considered the gold standard of clinical research [19] meaning their inclusion ensures a standard quality in result output, ruling out selection bias. To facilitate trial identification in OVID Embase and OVID Medline, a search filter for RCTs, created by the Scottish Intercollegiate Guidelines Network (SIGN) was included as part of the search strategy [20].

A UK search filter created by Ayiku et al was also included as part of the search strategy to ensure selected studies were performed in the region [21]. Year limits were used to ensure studies retrieved were published between 1^st^ December 2019 and 1^st^ January 2022.

### Inclusion and exclusion criteria

Studies that provide (A) participant demographics and (B) full eligibility criteria in the trial paper or supplementary materials were included in this review. Individual trial protocol papers, observational studies, systematic reviews, editorials, meta-analyses, reviews, and commentaries were excluded. Studies involving individuals under the age of 18 were excluded. Other reasons for exclusion were publication in a language other than English and studies conducted outside of the UK.

Based on these criteria, screening of the retrieved publications was conducted in stages using Covidence, which was also used to remove duplicates. Two reviewers independently screened the papers by both title and abstract; following this, papers were screened by their full text.

### Data extraction

From the publications included in this review, the following data were extracted: number of participants randomised, number of participants analysed, number of Black adult participants and number of adult minority ethnicity participants. Individual studies and their associated supplementary materials were then thoroughly assessed. Consequently, this led to the further extraction of: the study phase, participant demographics, recruitment methods and study site location.

### Statistical analysis

The extracted data from each publication was used to calculate the percentage of Black adults and adult ethnic minorities analysed in each study, using the overall number analysed denominator. A weighted average was also calculated to ensure study size did not inadvertently affect results. Following this, a two-tailed, one-sample t-test was conducted to statistically compare the sample means of our study data against the population mean, defined by the 2011 census data. *P* values < 0.05 were interpreted as statistically significant. Data was imported into R version 4.2.0 for statistical analysis with plots created using GGPLOT2.

### Assessment of risk of bias

Assessing bias in studies ensures trials are of a sufficient standard to be included within this review. This was assessed in each study using the Cochrane risk-of-bias tool for randomised trials (RoB 2) [22]. Studies were rated as either low, medium or high risk of bias in each domain, as well as being given an overall score.

## Results

**Fig. 1:**
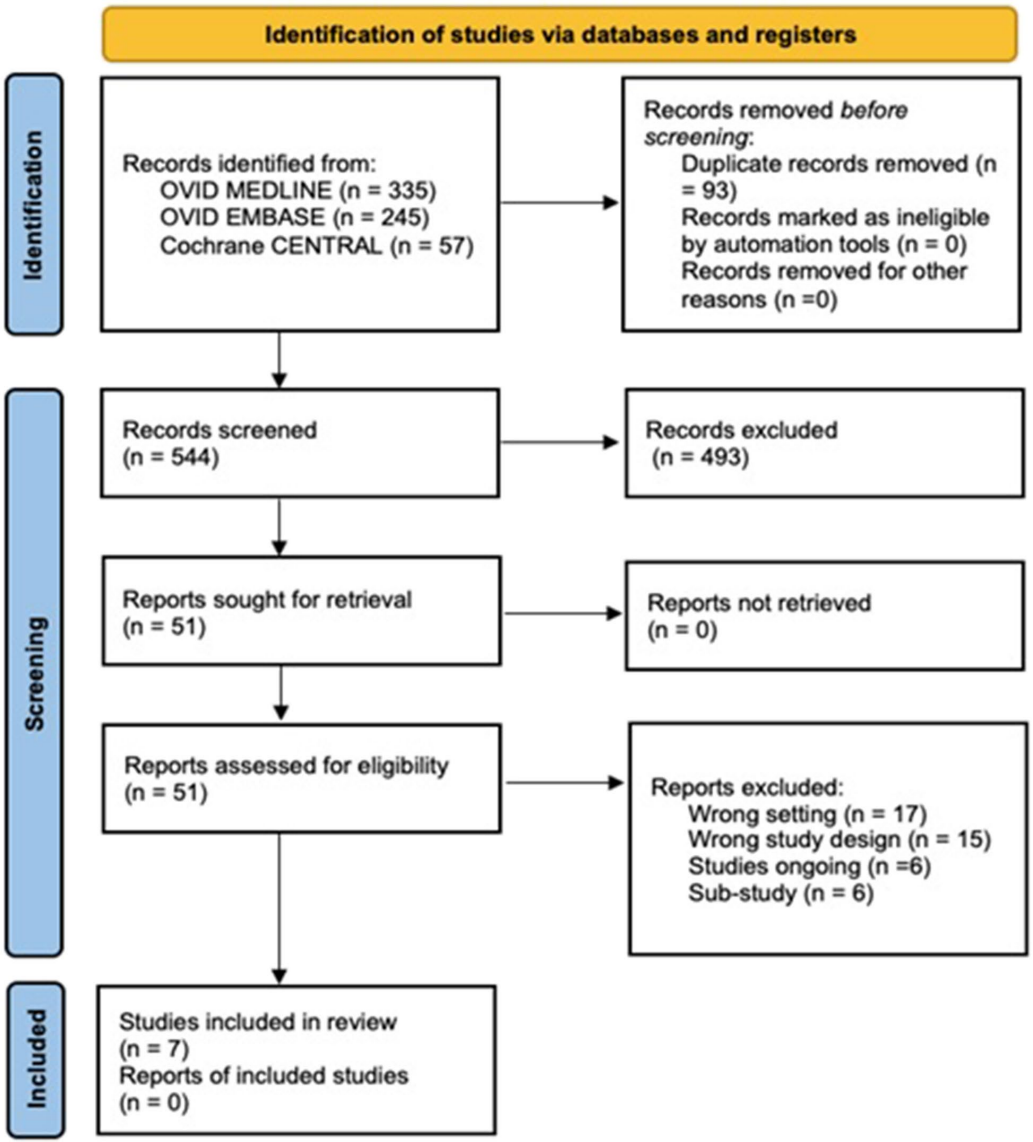
PRISMA flowchart of the study selection process

A total of 637 studies were identified from a search of OVID Medline, OVID Embase, and The Cochrane Central Register of Controlled Trials. After the removal of duplicates (n=93) and articles that did not align with the eligibility criteria by title, abstract and full text (*n*= 537), a sum of 7 articles have been included in this review [23–29] (Fig. 1).

### Study characteristics

All 7 studies in this review were conducted in the United Kingdom and published in English. Studies were published between 20^th^ July 2020 and the 2^nd^ of December 2021. The trial population of each study consisted of adults, however, age limits for recruitment varied between each publication. None of the trials reviewed clarified UK or British citizenship as a pre-requisite for recruitment. One study aimed to only recruit adults aged 30 and above [23]. Two studies recruited all adults aged 18 and above [24, 25]. Two studies focused on recruiting adults aged 50 and above [26, 27] and age limits were applied to the remaining two studies: ages 18 to 84 [28] and ages 18 to 55 [29] respectively. The trial phases also varied, and include phase 1/2 (*n*=1, 14%) [28], phase 2 (*n*=4, 57%) [23, 24, 26, 27] phase 3 (*n*=1, 14%) [28] and phase 4 (*n*=1, 14%) [25].

Enrolment in these studies took place between 23^rd^ April 2020 and 30^th^ June 2021, with the desired primary outcome of each being the determination of the safety, reactogenicity and immunogenicity of injectable vaccinations against COVID-19. The vaccines investigated in the selected publications are as follows: ChAdOx1 nCoV-19, AstraZeneca; BNT162b2, Pfizer–BioNTech; mRNA-1273, Moderna; NVX-CoV2373, Novavax; Ad26.COV2.S, Janssen; CVnCov, CureVac and VLA2001, Valneva.

### Outcome measures

A sum of 23,994 participants were enrolled to take part in COVID-19 vaccine RCTs at sites across the UK. Of this, ethnicity data was reported for 20,439 (85.2%) participants during either the enrolment or randomisation process. Raw data on participant demographics can be found in Table 1.

**Table 1:** Breakdown of raw values and the calculated prevalence of Black adults and adult ethnic minorities in the reviewed literature

Author	Number enrolled^**a**^	Number randomised ^**b**^	Number analysed^**c**^	Number (%) of Black adults^**d**^	Number (%) adult ethnic minorities^**e**^
Stuart et al	1072	1072	1072	13 (1.21%)	78 (7.28%)
Ramasamy et al	560	560	552	1 (0.18%)	28 (5.07%)
Folegatti et al	1077	1067	1077	6 (0.56%)	98 (9.10%)
Heath et al	16645	15187	14039	52 (0.37%)	718 (5.11%)
Lazarus et al	679	679	679	0 (0%)	25 (3.68%)
Liu et al	463	463	463	6 (1.30%)	117 (25.27%)
Munro et al	3498	2883	2557	13 (0.51%)	180 (7.04%)
Total	23994	21911	20439	91 (0.45%)	1244 (6.09%)

^a^ Total number of participants enrolled in the trial. ^b^ Total number of participants randomised in the trial. ^c^ Total number included for primary analysis. ^d^ Total number and percentage of Black adults randomised in the trial. ^e^ Total number and percentage of adult ethnic minorities randomised in the trial

### Primary outcome

Combining data from all 7 studies reviewed, 0.45% (91 of 20,439) of analysed participants identified as Black (Table 1). A weighted average based on trial size of Black adult participation (0.45%) was also calculated. The highest prevalence included in a single study to be 1.3%, (6 of 463) [26], with the lowest prevalence at 0% (0 of 679) [25] (Table 1).

**Fig. 2:**
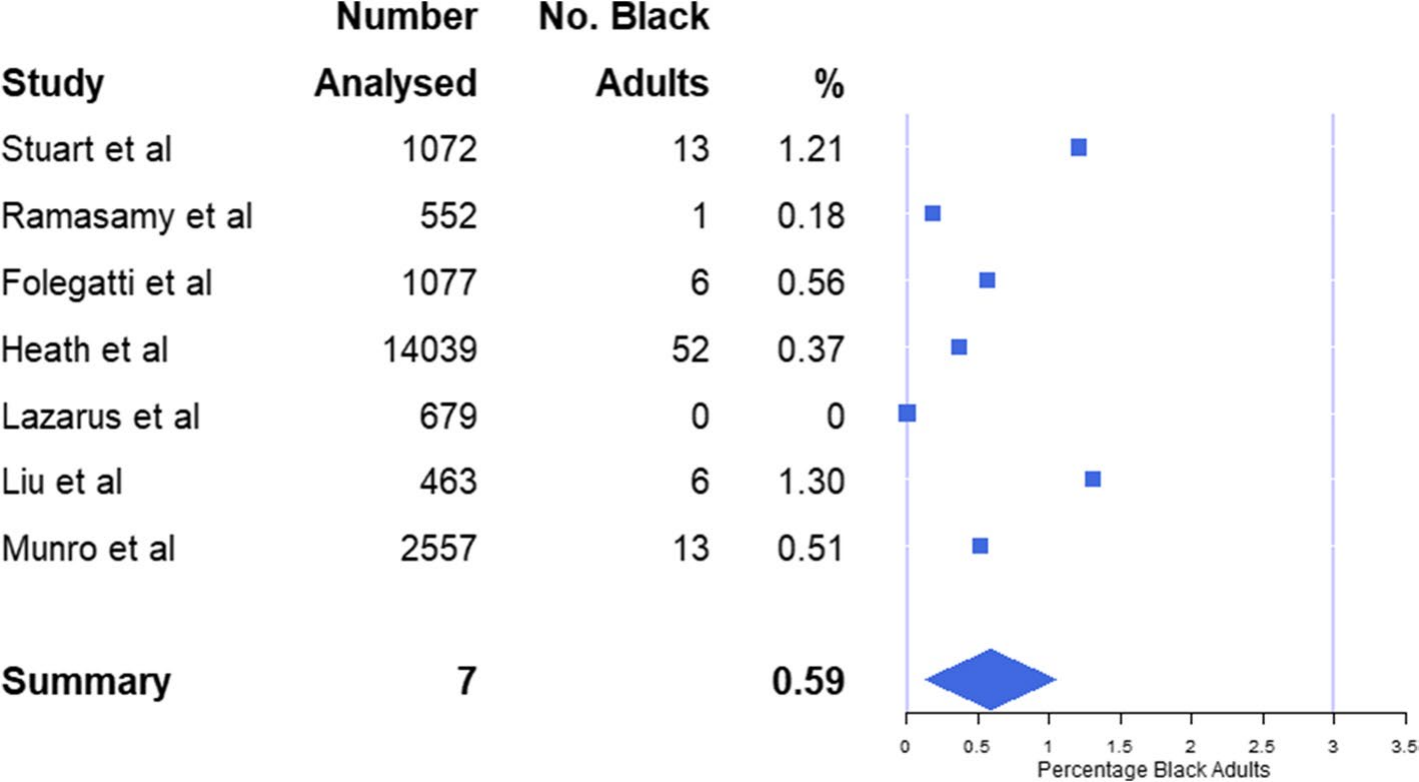
Plot with squares depicting the inclusivity (as a percentage) of Black adults across all 7 studies alongside a diamond depicting overall mean (with ends representing 95% CI) in comparison to the proportion they make up within the UK population (as represented by the line at 2.67%). NB: “No. black adults” denotes the number of Black adults enrolled in each study

A standard mean of 0.59% (95% CI: 0.13%, 1.05%) representation for all reviewed publications was calculated across all studies included (Fig. 2). With Black adults making up 2.67% of the UK adult population [15–17], a two-tailed, one-sample t-test comparing the mean representation against the 2011 census figure provides evidence (*p* < 0.001) that Black adults were under-served in UK-based COVID-19 vaccine RCTs.

### Secondary outcome

A total of 1244 (6.10%) adults who identified with a minority ethnic group (including Black), provided data for all 7 studies. The corresponding weighted average was 6.08%. The level of ethnic minority representation varied, with the highest prevalence being 25.27% (based on 117 of 463 randomised) [26] and the lowest being 3.68% (25 of 679) [25] (Table 1).

**Fig. 3:**
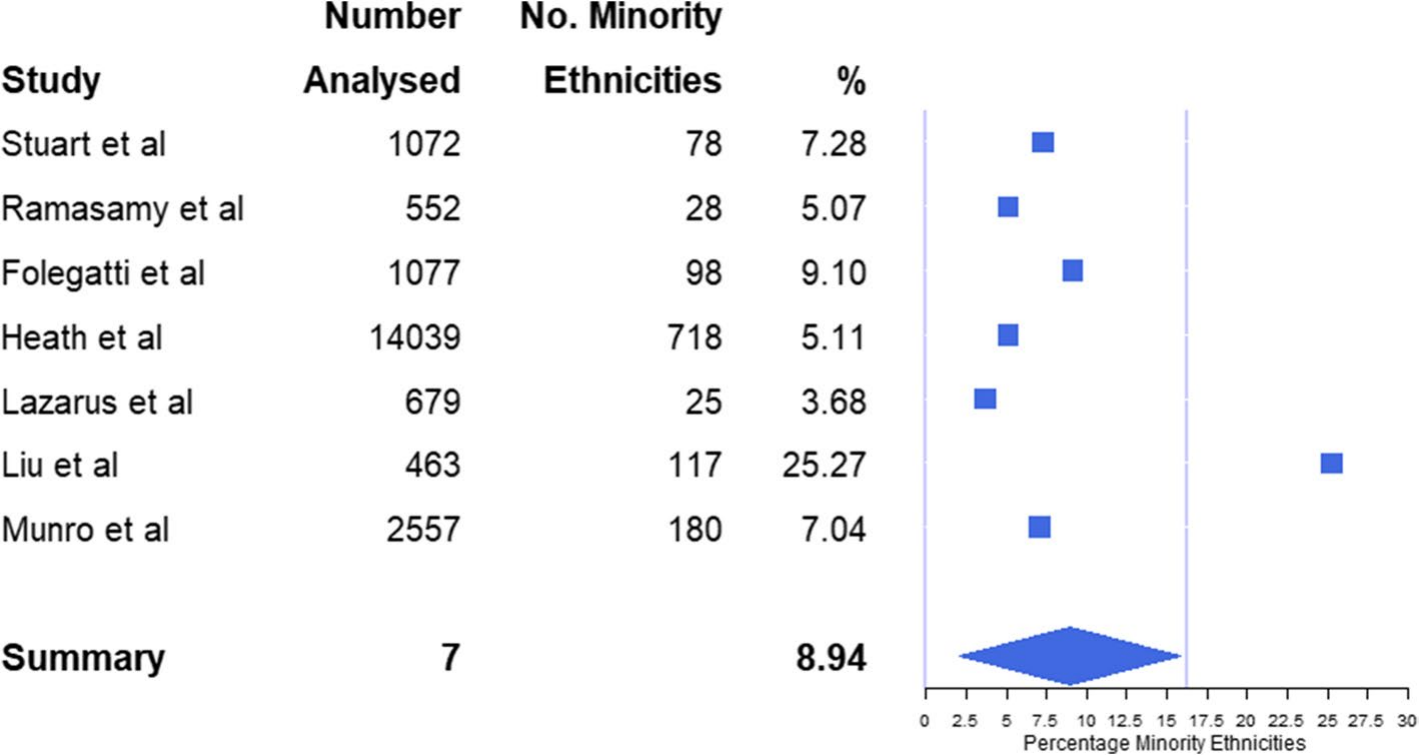
Plot with squares depicting the inclusivity of black adults and ethnic minorities (as a percentage) across all 7 studies alongside overall mean (with ends representing 95% CI), in comparison to the general population of the UK (as represented by the line at 16.30%)

A standard mean of 8.94% (95% CI: 2.07% - 15.80%) adult ethnic minority representation was calculated (Fig. 3). With adult ethnic minorities make up 16.30% of the UK population [17–19], a two-tailed, one-sample t-test comparing the mean representation against the 2011 census figure provides evidence (*p* = 0.039) that the minority ethnic group as a whole was under-served in UK-based COVID-19 vaccine RCTs.

### Inclusion and exclusion criteria

There was no identifiable exclusion of any ethnic groups when reviewing the inclusion and exclusion criteria of the studies. However, over half (*n*=4/7, 57%) listed insufficient English language level as grounds for exclusion [23, 26, 27, 29].

### Recruitment techniques

The recruitment methods used across each study remain relatively similar. All 7 studies utilised advertisements on social media as well as in public forums such as radio, newspapers and magazines to raise public awareness of the trial. Each study involved an online component of the recruitment and screening process, including forms to register interest in the trial. A major method for recruitment across all 7 studies - and the only recruitment method for one publication [28] - included email distribution to individuals who have already given consent to be contacted for any clinical trial at any trial sites. This includes databases such as the NHS COVID-19 online vaccine research registry, the Oxford Vaccine Centre databases and the NIHR COVID-19 vaccine volunteer database. Other recruitment techniques included direct mail out via the most recent electoral roll and using local GP practises or Trusts as participant identification centres.

### Study locations

**Fig. 4:**
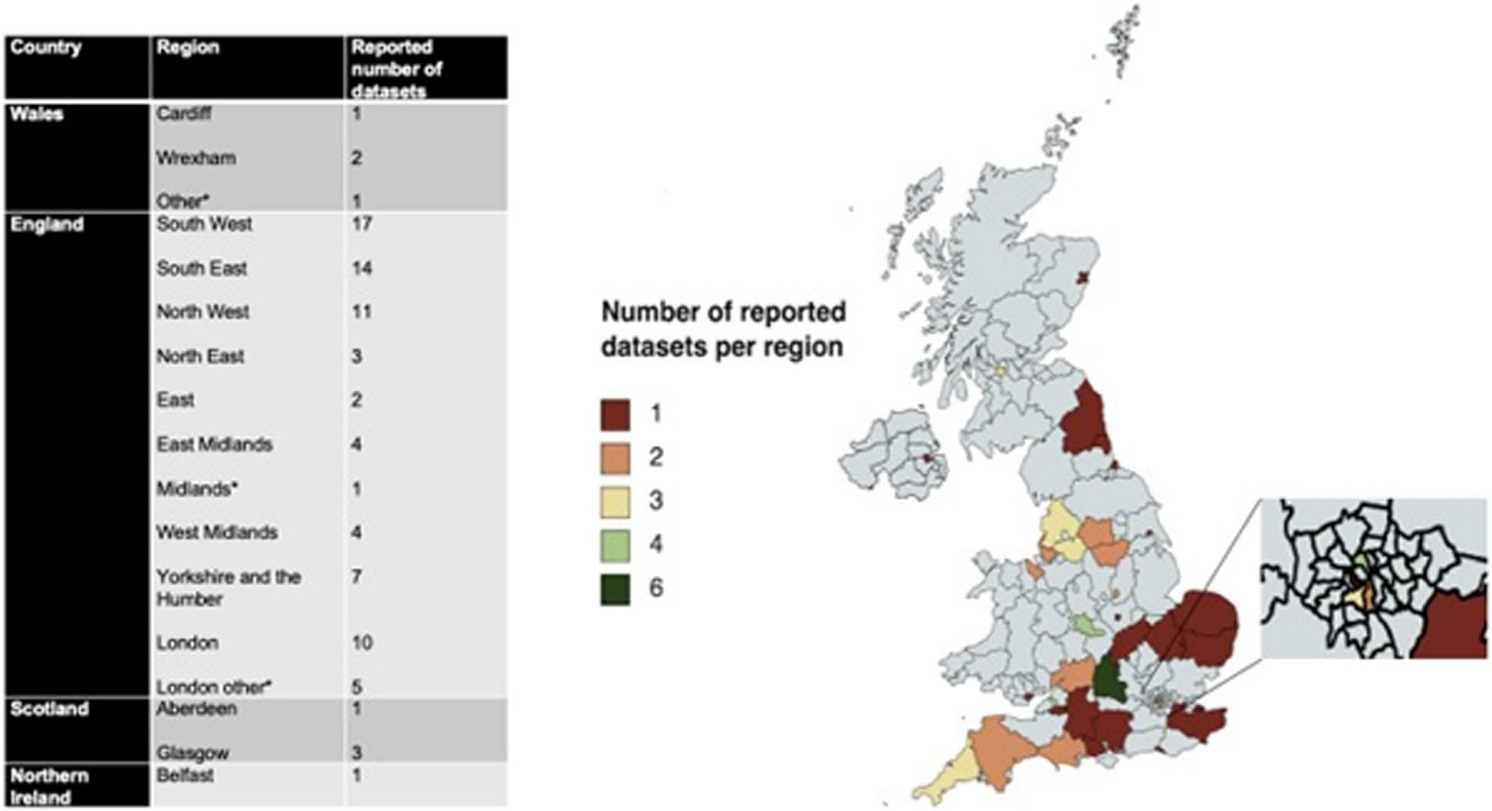
Number of reported datasets per region. *denotes where location was not able to be mapped

To provide some potential context to our primary and secondary outcome findings we have investigated the site locations of the 7 studies reviewed. In total, 87 sets of data collated from clinical trial sites across the UK contributed to the studies included in our review. Of these, 1 was collected from Northern Ireland, 4 from Wales (0.6% black, 4.4% non-white), 4 from Scotland and 78 from England (Fig. 4 & Supplement Figure 1). The majority of datasets (*n*=17, 19.5%), reported came from sites located in the South-West of England (0.9% black, 4.6% non-white) with the East of England (2.0% black, 9.2% non-white) having the lowest number of reported datasets (*n*=2, 2.3%). The remaining datasets reported per region of the UK are as follows: London (*n*=15, 17.2%; 13.3% black, 40.2% non-white), South-East England (*n*=13, 15.0%; 1.6% black, 9.3% non-white), North-West England (*n*=11, 12.6%; 1.4% black, 9.8% non-white), Yorkshire and the Humber (*n*=7, 8.1%; 1.5% black, 11.2% non-white), East Midlands (*n*=5, 5.8%; 1.8% black, 10.7% non-white), West Midlands (*n*=4, 4.6%; 3.3% black, 17.3% non-white ) and North East England (*n*=3, 3.5%; 0.5% black, 4.7% non-white). Breakdowns of ethnicity by area were obtained from the 2011 census [15] with data for Scotland and Northern Ireland unavailable.

### Risk of bias

Using the RoB 2 tool, [22], each study was determined to have a low risk of bias. Whilst three studies did not provide outcome data for nearly all of the participants randomised [23, 27, 28] (determined as over 95%), the provision of an intention-to-treat or modified intention-to-treat analysis was considered sufficient evidence that this did not influence the study outcomes.

## Discussion

This systematic review included data from 20,437 individuals across 7 COVID-19 vaccine RCTs conducted in the UK. From the studies reviewed, the calculation of Black adult representation revealed a mean of 0.59%. This was significantly different than the population level of Black adults (2.67%) as per 2011 census data. A similar trend among the adult ethnic minority population (8.94%) which was significantly lower than the census data for other ethnic minorities (16.30%). This would suggest that both Black and ethnic minority adults were under-represented in COVID-19 vaccine RCTs.

One study from this review did show progress in ethnic minority recruitment. The data from Liu et al [26] indicates that adult ethnic minorities had been over-represented by 8.97%, however, this study only aimed to include participants aged 50 and above. When also considering 2 of the 8 study sites used in this trial were based in the South-West of England with a non-white population of <5% [15] you would anticipate the proportion of those enrolled defined as White British would be potentially higher than the national average, as they are the dominant demographic within this age group and location [15]. The success of diversity in the trial study population is explained within the paper via the utilisation of a wide range of recruitment techniques, including the distribution of advertisements in public places (a recommendation suggested by INCLUDE [14]). The study protocol (26, supplement), explicitly aims to encourage recruitment of “those identifying as Black, Asian and Minority Ethnic”. It should also be noted that, of the 8 study sites, 7 were located in highly populated UK cities which are commonly associated with higher proportions of ethnic minority populations [15]. The study which failed to recruit any Black adults [25] covered 12 sites, 9 of which were in the South-West. The only site with located in an area with an above-average Black population (London) only recruited 15 (2.2%) of patients.

Outside of Liu et al, studies failed to show representative enrolment. Overall, 17.24% of participant demographics retrieved for this review were obtained from sites located in London (15 of 88 sites assessed, Supplement Figure 1), with a Black population of 13.3% [15]. The overall low representation of Black adults suggest a failure to engage with minority ethnicities in areas where there is a sufficient pool to recruit from. Likewise, a substantial proportion of participants randomised were from sites based in regions with the lowest representation of minority ethnicities. For example, South-West England (*n*=17) (Fig. 4), only has a Black African population of 0.9%, and an overall minority ethnic population of 4.7% [30]. Whilst it is appreciated that trial location is dependent on variables such as available staffing and funding, performing trials in areas with little minority representation will ensure they remain an under-served group.

Another key aspect of our findings identified the potential importance of inclusive recruitment techniques. Using INCLUDE guidance [14] in conjunction with utilising sites in ethnically diverse areas would be anticipated to have a positive effect on recruitment. However, none of the manuscripts reviewed made specific reference to INCLUDE. However, three of the studies specifically identified Black, Asian and minorities ethnic groups as a priority for recruitment [23, 26, 27] although none utilised patient and public involvement (PPI) groups in their study design to aid this (except for in dissemination of results [25]). Four of the studies included in our review contained insufficient English language level as part of their exclusion criteria [23, 26, 27, 29]. This can act as a deterrent for ethnic minorities - a group which also encompasses migrants, who are more likely to have a poor understanding of a host country’s health system and language [31]. Recruitment via email distribution to individuals who have already given consent to be contacted for clinical trials was identified across all 7 studies. One study primarily recruited participants from the NHS COVID-19 volunteer database [28]; however, only 0.7% of volunteers registered on this server identify as Black, African, Black British or Caribbean [32]. Recruiting primarily from this source dictates that the overall nature of results will not be generalisable as this demographic profile is not reflective of the UK population. This is also contradictory to INCLUDE guidelines [14], which instruct researchers to recruit participants through as wide a range of means as possible.

Online screening processes are associated with all 7 of our studies, where potential participants are asked to report details such as medical history. Whilst an online platform provides an appropriate tool of contact during national and local lockdowns, research has suggested that online and virtual consultations amplify existing inequalities in access to healthcare for many migrants due to a lack of digital literacy and access to technology [33]. All of this solidifies their standing as an under-served group in clinical research, in addition to leaving them at risk of misinformation around vaccines due to a lack of communication with health services.

It is appreciated that more inclusive recruitment processes are difficult to attain, especially in the context of the COVID-19 pandemic where timings for trial set-up and recruitment were vastly reduced, and sufficient public involvement was harder to achieve. Strategies such as the provision of a translator with scientific knowledge in the appropriate language can incur an additional large cost on the trial funders, as well as taking longer to perform [34]. However, language as part of eligibility criteria arguably reduces external validity by creating heterogeneity of participants, where English-speaking participants are over-represented in relation to the overall UK population and so become the standard. Therefore, appropriate steps should be taken to improve inclusivity in research, including the provision of translators to improve the inclusion of under-served groups. Furthermore, this respects NIHR guidelines stating that every eligible person who wishes to take part in research, regardless of background, should be offered the same opportunity [14]. We subsequently recommend that trials not only follow INCLUDE guidance [14], but to refer to the document and explicitly state any guidance taken within trial documentation.

For future research, it would be useful to have corresponding demographic output of excluded participants to establish whether discrepancies in inclusivity are due to design (especially exclusion criteria) or down to an overall failure to recruit. A deeper dive into urban/rural breakdown by site location is possible. Likewise, an assessment of recruitment methods and the extent to which trials follow INCLUDE guidance [14] would assist in steering future conversation on inclusivity in recruitment.

## Conclusion

Black adults, and adult ethnic minorities, remain under-served in COVID-19 clinical research, despite being disproportionately affected by the disease. This highlights the importance of projects like INCLUDE [14], which has an invaluable role in raising awareness of under-served groups in clinical research to adequately improve the inclusivity of these groups within future vaccine RCTs in the UK as per population estimates. Without inclusive trial populations, results may not be generalisable and thus the external validity is limited and threatened. Furthermore, the exclusion of under-served groups puts them at risk of worse health outcomes due to the risk of deployment of interventions and therapeutics that are ineffective (or even harmful) to some of the population [35]. As such, more inclusive practices must be developed and widely shared in the recruitment of underserved groups, as it is key to understanding the true impact of COVID-19 on the most vulnerable groups of society, and the population as a whole.

NB: “No. minority ethnicities” denotes the number of minority ethnicity adults enrolled in each study

### Supplementary Information

**Additional file 1:** **Supplement Figure 1.** Bar chart depicting the number of reported datasets per region of the UK. * represents where some specific trial site locations were unavailable within this region

**Additional file 2.**

## Data Availability

All data relevant to the study are included in the article or uploaded as supplementary information.
