# Supplementary material for: How inclusive were UK-based randomised controlled trials of COVID-19 vaccines? A systematic review investigating enrolment of Black adults and adult ethnic minorities
Source: Trials. 2024 Apr 12;25:255. doi: 10.1186/s13063-024-08054-4 (PMC11010339; doi:10.1186/s13063-024-08054-4)
Supplement: Supplementary file 1 — Additional file 1: Supplement Figure 1. Bar chart depicting the number of reported datasets per region of the UK. * represents where some specific trial site locations were unavailable within this region [file 13063_2024_8054_MOESM1_ESM.zip › HHNJ_TRIALS_JUN23_supplementR2.docx]

**SUPPLEMENTARY MATERIAL**

**How inclusive were UK-based randomised controlled trials of COVID-19 vaccines? A systematic review investigating enrolment of Black adults and adult ethnic minorities**

Hibba Herieka^1^, Daphne Babalis^2^, Evangelia Tzala^3^, Shyam Budhathoki^4^, Nicholas Johnson^2^

^1^ University of Leicester Medical School, University of Leicester, Leicester, United Kingdom

^2^ Imperial Clinical Trials Unit, School of Public Health, Faculty of Medicine, Imperial College London, London, United Kingdom

^3^ School of Public Health, Faculty of Medicine, Imperial College London, London, United Kingdom

^4^ Department of Primary Care and Public Health, School of Public Health, Faculty of Medicine, Imperial College London, London, United Kingdom

^*^ Corresponding Author

Nicholas A Johnson, Imperial Clinical Trials Unit (ICTU), Stadium House, 68 Wood Lane, London, W12 7RH; nicholas.johnson@imperial.ac.uk

**Supplement Figure 1 – Bar chart depicting the number of reported datasets per region of the UK.**

*** represents where some specific trial site locations were unavailable within this region**
