# Supplementary figures and images for: How inclusive were UK-based randomised controlled trials of COVID-19 vaccines? A systematic review investigating enrolment of Black adults and adult ethnic minorities
Source: Trials. 2024 Apr 12;25:255. doi: 10.1186/s13063-024-08054-4 (PMC11010339; doi:10.1186/s13063-024-08054-4)

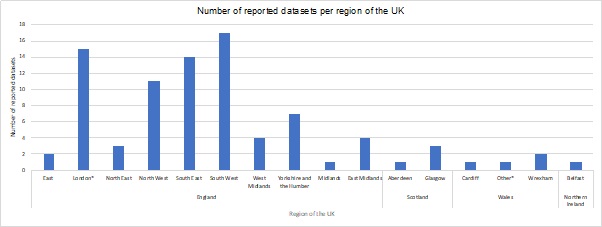

Supplement: Supplementary file 1 — Additional file 1: Supplement Figure 1. Bar chart depicting the number of reported datasets per region of the UK. * represents where some specific trial site locations were unavailable within this region [file 13063_2024_8054_MOESM1_ESM.zip › Supplement Figure 1R2.jpg]
